# Changes in Physical Function and Effects on QOL in Patients after Pancreatic Cancer Surgery

**DOI:** 10.3390/healthcare9070882

**Published:** 2021-07-13

**Authors:** Hideaki Kurokawa, Yoshiteru Akezaki, Ritsuko Tominaga, Masaki Okamoto, Masato Kikuuchi, Makiko Hamada, Yoshihiro Mikuriya, Koji Ohta, Shinsuke Sugihara

**Affiliations:** 1 Department of Rehabilitation Medicine, National Hospital Organization Shikoku Cancer Center, Ehime 791-0280, Japan; kurokawa.hideaki.ac@mail.hosp.go.jp (H.K.); tominaga.ritsuko.fk@mail.hosp.go.jp (R.T.); okamoto.masaki.cb@mail.hosp.go.jp (M.O.); kikuuchi.masato.tu@mail.hosp.go.jp (M.K.); sugihara.shinsuke.rk@mail.hosp.go.jp (S.S.); 2 Division of Physical Therapy, Kochi Professional University of Rehabilitation, Kochi 781-1102, Japan; 3 Department of Rehabilitation Medicine, Higashi Tokushima Medical Center, Tokushima 779-0193, Japan; hamada.makiko.wu@mail.hosp.go.jp; 4 Department of Gastroenterological Surgery, National Hospital Organization Shikoku Cancer Center, Ehime 791-0280, Japan; mikuriya.yoshihiro.pn@mail.hosp.go.jp (Y.M.); ota.koji.zh@mail.hosp.go.jp (K.O.)

**Keywords:** physical function, quality of life, pancreatic cancer, surgery, rehabilitation

## Abstract

This study examined the changes in physical function and quality of life (QOL) of postoperative patients with pancreatic cancer for 3 months after surgery and examined the factors affecting the QOL at the 3 months after surgery. Methods: This study comprised 32 pancreatic cancer patients who underwent surgery at our hospital. Among these patients, 20 patients for whom data was measured before surgery to 3 months after surgery were selected for statistical analyses: 8 males and 12 females, 69.8 ± 7.4 years. The preoperative and postoperative rehabilitation was given to patients under the guidance of a physiotherapist. Nutritional status, body composition, physical function, gait assessments, and QOL were investigated. Results: Body weight, body fat mass, body fat percentage, body mass index (BMI), and muscle mass significantly decreased 3 months after surgery compared with their respective preoperative values. The mean grip strength at the time of 3 months after the surgery had decreased significantly from 27.3 kg to 24.5 kg. The mean skeletal muscle mass index (SMI) had decreased significantly from 6.3 kg before surgery to 5.9 kg after the surgery. The QOL scores for global health status, physical, and role showed significant decreases 2 weeks after surgery compared with the respective preoperative scores. Significant improvements in these scores were observed 3 months after surgery compared with the respective scores 2 weeks after surgery. Physical function assessments after surgery were associated with QOL 3 months after surgery. Conclusion: Recovery of patients after pancreatic cancer surgery in body weight, BMI, body fat percentage, body fat percentage, muscle mass, SMI, and grip strength was not sufficient at the time of 3 months after surgery. It has been observed that physical function of patients has affected the improvement of QOL.

## 1. Introduction

Pancreatic cancer is an aggressive malignancy and one of the leading causes of cancer-related death [1,2]. Although surgical resection is an effective treatment for pancreatic cancer, many patients experience recurrence after surgery [1,2].

Pancreatic cancer patients have experienced reduced physical function, such as loss of endurance and muscle strength [3]. Body weight loss and sarcopenia may also occur and are poor prognostic factors for postoperative patients with gastrointestinal cancer [4,5]. Pancreatic cancer survivors have been reported to experience reduced quality of life (QOL), including diminished physical and psychological well-being, compared with the survivors of other malignancies and individuals without cancer [6]. Treatment to maintain physical function and improve QOL is very important due to the poor prognosis of patients with pancreatic cancer [7].

In patients with esophagogastric cancer, physical function can also be significantly impaired during recovery, both pre-discharge [8] and 3 months post-surgery [9]. Few reports have examined changes in the physical functions of patients after pancreatic cancer surgery over time and the effects of physical function on QOL. Examining how physical function affects QOL in postoperative patients with pancreatic cancer can be useful for determining the need for interventions to improve QOL.

This study examined the changes in physical function and QOL of postoperative patients with pancreatic cancer for 3 months after surgery and examined the factors affecting the QOL at the 3 months after surgery.

## 2. Methods

### 2.1. Patients and Methods

This study comprised 32 pancreatic surgery patients who underwent surgery at our hospital between April 2018 and March 2020. Among these patients, 20 patients for whom data was measured before surgery to 3 months after surgery were selected for statistical analyses.

Patients fulfilling the following criteria were excluded: (1) Barthel index < 100 scores, (2) Revised Hasegawa Dementia Scale (HDS-R) ≤ 20 scores, (3) patients who were not available for evaluation before, 2 weeks, 1 month, and 3 months after surgery.

Nutritional status (albumin), body weight, body mass index (BMI), body fat mass, body fat percentage, muscle mass, skeletal muscle mass index (SMI), grip strength, muscle strength of the lower limb, one-leg standing time, gait, and QOL were investigated. Measurements were performed before the surgery and 2 weeks, 1 month, and 3 months after surgery.

### 2.2. Rehabilitation Program

The preoperative program consisted of muscle-strengthening exercises—squatting and calf raise exercises—and walking. The preoperative program was performed for 2 days before surgery. On postoperative day (POD) 1, sitting, standing, and walking still were started. On POD 4, muscle-strengthening exercises were started. On POD 7, aerobic exercise, stair climbing/descending and outdoor walking were performed, and physical activities were increased depending on the patient’s physical condition. In food textures, POD 4, controlled diet (rice gruel in three degree) was started. Starting POD 7 after the operation, the subjects of this study were allowed to consume a normal diet. The timing of mobilization changed according to the physical status of the patient for a few days.

Squatting and calf raise exercises were performed 20 times each using the patient’s own body weight. The preoperative and postoperative rehabilitation were 20 min per day. Exercise intensity for preoperative and postoperative rehabilitation were set at 3–5 points on the Borg’s Category-Ratio scale (Borg CR-10). The preoperative and postoperative were performed under the guidance of a physiotherapist.

At discharge, the physiotherapist instructed patients to perform the same rehabilitation performed during hospitalization at home. For meals as well, patients were instructed on the contents of meals after discharge, how to eat, and how to drink water.

### 2.3. Body Composition

BMI, body fat mass, body fat percentage, muscle mass, and SMI were measured by bioimpedance analysis using In Body S720 (In Body Japan Inc., Tokyo, Japan) [10]. The measurement was performed after 1 h or more after eating. SMI was evaluated by dividing the limb skeletal muscle mass (kg) by the square of the height (m^2^).

### 2.4. Physical Function

Handgrip strength was measured using a digital dynamometer (TTM, Inc., Tokyo, Japan), with the participant standing upright [11]. The patients had shoulder adducted and neutrally rotated, elbows fully extended, forearms and wrists in neutral position, and legs open to shoulder width. The patients needed to keep the dynamometer away from any part of the body.

To measure the muscle strength of the lower limb, the quadriceps muscle strength was measured using a hand-held dynamometer (ANIMA, μ-Tas F-01) [12]. Measurements were made with the patients sitting upright on a treatment bed, without back support, and with both upper extremities crossed in front of the trunk. Patients sat with the knee joint at 90-degree flexion. The sensor pad of the hand-held dynamometer was attached to the front of the distal crus. The sensor was fastened using Velcro placed just above the malleolus and the sensor pad was affixed to a belt connected to a pillar of the treatment bed. The patients were then asked to make a maximum isometric contraction of the quadriceps for 5 s. The recorded value (kgf) was divided by the patient’s body weight, and this value (kgf/kgf) was defined as the muscle strength of the lower limb.

For the one-leg standing time, the patients were asked to stand on one leg at a time, with their eyes open and both hands on their hips [13]. The measurement ended when the patients were no longer able to maintain balance and the suspended leg touched the floor, or the position of the supporting lower limb shifted. The one-leg standing time was measured using a stopwatch. The maximum achievable one-leg standing time was 60 s.

Grip strength, muscle strength of the lower limb, and one-leg standing time were measured twice on each side, and the maximum values were used.

### 2.5. Gait Assessments

For gait assessments, 5 m fast gait speed was measured [14]. The time required to walk the middle 5 m of a 7 m, indoor, flat surface was recorded. The patients were asked to walk as quickly and safely as possible without running. Patients performed the test twice, and the lowest time registered on stopwatch was considered for the study.

### 2.6. Quality of Life

The EORTC-QLQ-C30 is a questionnaire for QOL measure, designed for use in cancer patients. The QLQ-C30 includes a global health status item, five functional scales (physical, role, emotional, cognitive, and social), three symptom scales (fatigue, nausea or vomiting, and pain), and six single-item symptom measures (dyspnea, insomnia, appetite loss, constipation, diarrhea, and financial difficulties) [15]. In this study, among the items offered in the QLQ-C30, the global health status item and the five functional scales (physical, role, emotional, cognitive, and social) were used.

### 2.7. Statistical Analysis

For pre- and postoperative comparisons of nutritional status body composition, physical function, gait assessment, and QOL, repeated-measures analysis of variance was applied to identity the differences among before, 2 weeks, 1 month, and 3 months after surgery, and then differences between periods of evaluation were compared with Tukey’s test. The Friedman test was administered to analyze differences among preoperative, 2 weeks, 1 month, and 3 months after surgery, and then the Wilcoxon signed-rank test with Bonferroni correction was applied. The choice of the repeated-measures analysis of variance or Friedman test was made according to the result of the Shapiro–Wilk normality test.

To assess which factors affect QOL 3 months after surgery, stepwise multiple regression analyses were performed, using the items from the QLQ-C30 (global health status, physical, role, emotional, cognitive, and social) as dependent variables and age, sex, albumin, weight, BMI, body fat mass, body fat percentage, muscle mass, SMI, grip strength, muscle strength of the lower limb, one-leg standing time, and gait assessment data from 3 months after surgery as the independent variables. SPSS software version 22.0 (IBM Corp., Armonk, NY, USA) was used to analyze the collected data. The significance level was set to *p* < 0.05 for repeated measures analysis of Variance and Friedman’s tests, and the Wilcoxon signed-rank results were used to adjust the *p*-values for multiple pairwise comparisons (*p* < 0.05/6 = 0.008, corrected for 6 pairwise comparisons) for the test after the Friedman’s tests.

## 3. Results

### 3.1. Socio-Demographic and Clinical Characteristics

Table 1 shows characteristics of the study patients. Operative procedures were pancreaticoduodenectomy in 3 patients, subtotal stomach-preserving pancreatoduodenectomy in 6 patients, pylorus-preserving pancreatoduodenectomy in 1 patient, and distal pancreatectomy with celiac axis resection in 10 patients. The mean volume bleeding during surgery was 803.8 ± 425.6 mL. The mean period of postoperative rehabilitation was 14.7 ± 5.8 days. No patients had exocrine pancreatic insufficiency. After discharge, no specific rehabilitation was given to patients at the hospital.

### 3.2. Differences in Nutritional Status, Body Composition, Physical Function, and Gait Assessment before, Two Weeks, One Month, and Three Months after Surgery

**Figure 1:**
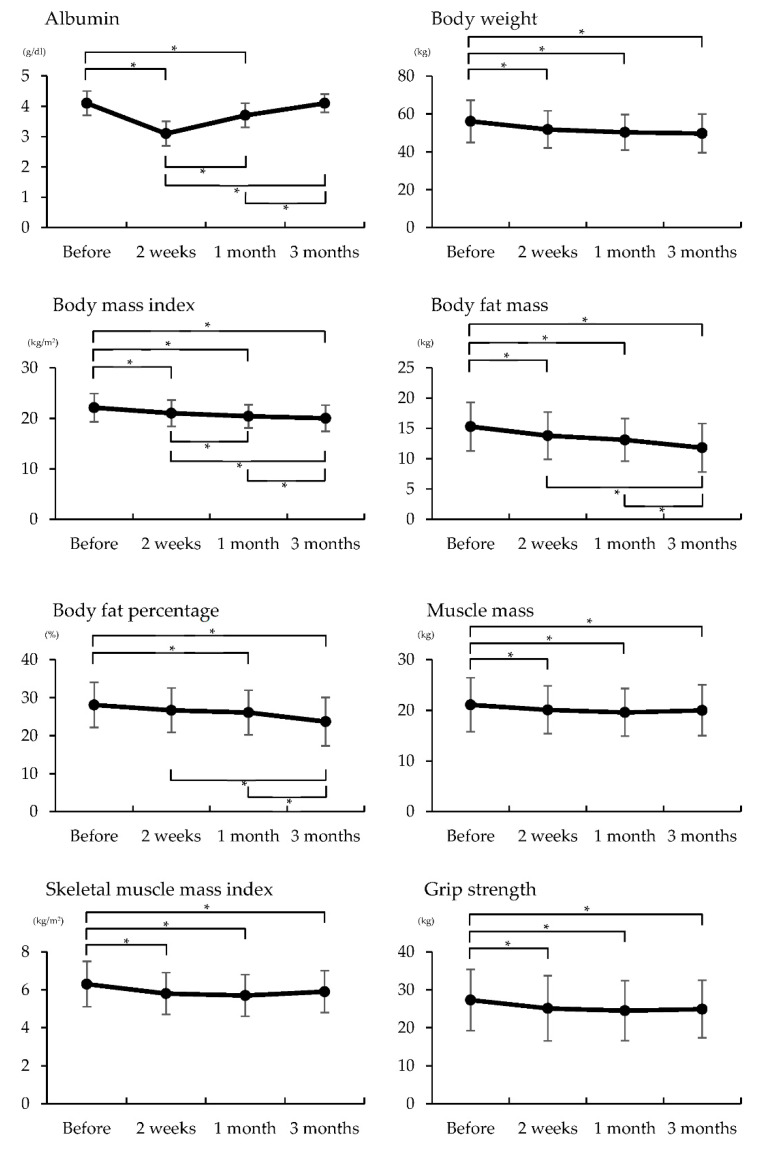

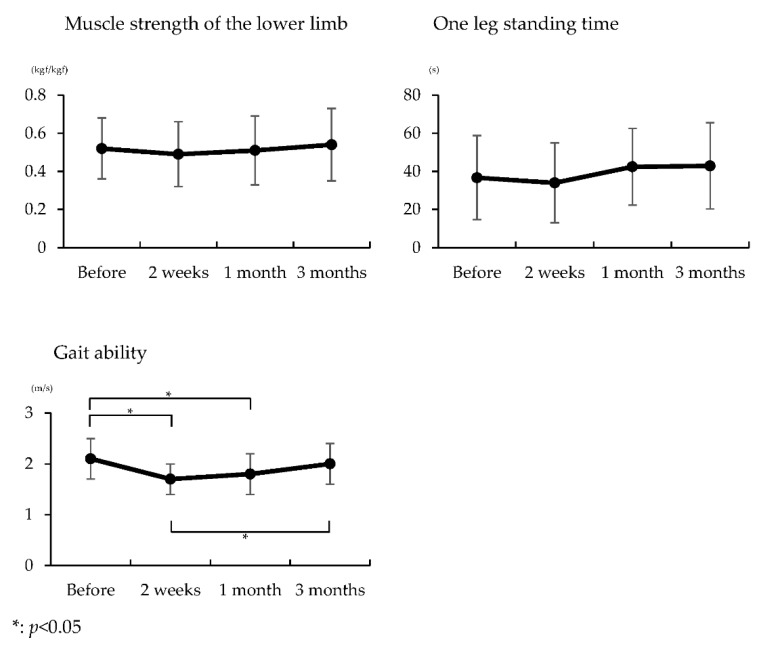
Comparisons of body composition, physical function, and gait ability at before, two weeks, one month, and three months after surgery.

The results of albumin, body composition, physical function, and gait assessment before, 2 weeks, 1 month, and 3 months after surgery are shown in Figure 1. Albumin was significantly reduced at 2 weeks and 1 month after surgery compared with preoperative albumin (*p* < 0.05). The level of albumin 2 weeks after surgery was significantly lower than 1 month and 3 months after surgery (*p* < 0.05). The level of albumin 3 months after surgery was significantly higher than 1 month after surgery (*p* < 0.05). Body weight was significantly reduced at 2 weeks, 1 month, and 3 months after surgery compared with preoperative body weight (*p* < 0.05). BMI also decreased significantly 2 weeks, 1 month, and 3 months after surgery compared with preoperative BMI (*p* < 0.05). BMI values at 1 month and 3 months after surgery were significantly lower than BMI at 2 weeks after surgery (*p* < 0.05). No significant difference was observed between BMI values at 1 and 3 months. Body fat mass was significantly reduced at 2 weeks, 1 month, and 3 months after surgery compared with preoperative values. Body fat percentage was significantly reduced at 1 month and 3 months after surgery compared with preoperative values. The level of body fat mass and body fat percentage 3 months after surgery were significantly lower than 2 weeks and 1 month after surgery (*p* < 0.05). Muscle mass, SMI, and grip strength showed significant decreases at 2 weeks, 1 month, and 3 months after surgery compared with the respective preoperative values (*p* < 0.05). Gait speed showed a significant deterioration at 2 weeks and 1 month after surgery compared with the value before surgery, and the gait at 3 months after surgery was significantly improved compared with the gait at 2 weeks after surgery (*p* < 0.05).

Three patients were diagnosed with sarcopenia at 3 months after surgery according to parameters by Cruz-Jentoft et al. (2019).

### 3.3. Differences in Quality of Life Scores before, Two Weeks, One Month, and Three Months after Surgery

**Figure 2:**
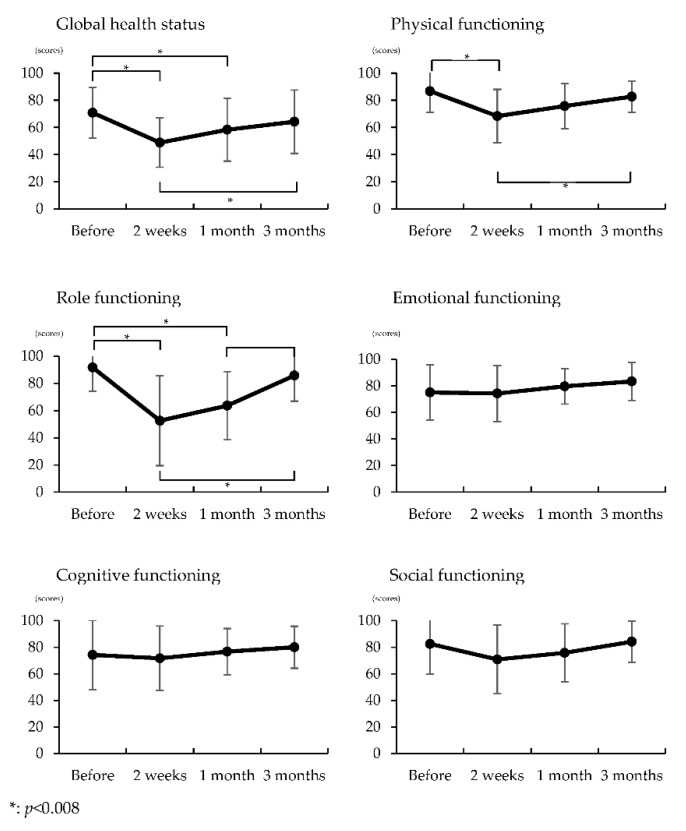
Comparisons of quality of life at before, two weeks, one month, and three months after surgery.

The QLQ-C30 results before, 2 weeks, 1 month, and 3 months after surgery are shown in Figure 2. The global health status score showed a significant decrease at 2 weeks and 1 month after surgery compared with the preoperative score. The score at 3 months was significantly improved compared to the score at 2 weeks (*p* < 0.05). The QOL physical score showed a significant decrease at 2 weeks after surgery compared with the preoperative score. The score at 3 months was significantly improved compared with the value at 2 weeks (*p* < 0.05). The QOL role score showed a significant decrease at 2 weeks and 1 month after surgery compared with the preoperative score (*p* < 0.05). The score 3 months after surgery was significantly improved compared with the scores 2 weeks and 1 month after surgery (*p* < 0.05).

### 3.4. Factors Predicting Quality of Life at Three Months after Surgery

Stepwise multiple regression analyses is shown in Table 2. The grip strength 3 months after surgery was associated with global health status score (*p* < 0.05, R^2^ = 0.322).

## 4. Discussion

This study aimed to clarify the changes in physical function and QOL among postoperative patients with pancreatic cancer and determine the factors that affect QOL. The results showed that body composition and grip strength significantly decreased 3 months after the operation compared with the respective preoperative values. The QOL scores for global health status, physical, and role showed significant decreases 2 weeks after surgery compared with the respective preoperative scores, and significant improvements in these scores were observed 3 months after surgery compared with the respective scores 2 weeks after surgery. Physical function assessment after surgery was associated with QOL 3 months after surgery.

After pancreatic resection, patients showed significantly reduced cardiorespiratory fitness (VO_2peak_), functional capacity (6-min walking distance), and muscle strength compared with healthy controls [3]. Another study demonstrated that 6 months of progressive resistance training improved muscle strength [16]. A home walking program resulted in significant improvements in resected pancreas and periampullary cancer patients in terms of fatigue levels, physical function, and health-related QOL scores [17]. In this study, we promoted early mobilization after surgery and performed rehabilitation, such as muscle strengthening exercises and ADL exercises for an average of 15 days after surgery, while only the patient’s own self-training was performed after discharge. In this study, SMI and grip strength decreased significantly compared with the preoperative values, even 3 months after surgery. Sarcopenia is diagnosed based on the detection of low muscle mass and low muscle function (muscle strength or physical performance) [18], and sarcopenia has been significantly associated with poorer overall survival [19]. Three patients in this study were diagnosed with sarcopenia, and patients with sarcopenia may increase in the future. Additionally, decreased physical function can result in decreased ADL.

Body weight loss is associated with reduced physical function [19], lower QOL, reduced tolerance to anticancer therapy [4], and overall survival [5,20]. In this study, 4 days after the operation, controlled diet (rice gruel in three degree) was started. Additionally, starting 7 days after the operation, the subjects of this study were allowed to consume a normal diet and did not have any dietary restrictions. At the time of discharge, we provided nutritional guidance at home. In this study, body weight, body fat mass, body fat percentage, and BMI decreased significantly, even 3 months after surgery compared with their respective preoperative values. On the other hand, albumin showed improvement 3 months after the surgery. Reduction of Dietary intake of patients with pancreatic cancer is affected by the effects of surgery, it is considered that the lack of nutritional guidance after discharge have affected the results. Results of this study suggests that patients need continuous nutritional management even after discharge.

After pancreatic cancer surgery, patients have been reported to experience a significant decline in QOL 14 days after resection [21]. QOL 60 days after surgery was reported to be significantly worse than preoperative QOL, although emotional functioning was found to be significantly better after surgery than preoperatively [21]. In contrast, another study showed comparable QOL scores between measurements performed preoperative and measurements performed 3 and 6 months after surgery [22]. In this study, the global health status, physical, and role scores decreased 2 weeks after surgery and improved 3 months after surgery. Grip strength at 3 months after surgery was found to be factors affecting global health status QOL scores. The results of this study showed that patients with pancreatic cancer must actively perform postoperative rehabilitation.

### Study Limitations

First, the number of subjects was small. Second, all subjects in this study underwent rehabilitation interventions and were not compared with a non-active control group. Third, the patient’s home exercise time, physical activity, and dietary status could not be evaluated after discharge. Fourth, we could not clarify the factors that affected emotional, cognitive, and social QOL scores. Further consideration of these factors remains necessary in the future.

## 5. Conclusions

This study aimed to clarify the changes in physical function and QOL among postoperative patients with pancreatic cancer and determine the factors that affect QOL.

Recovery of patients after pancreatic cancer surgery in body weight, body mass index, body fat percentage, body fat percentage, muscle mass, SMI, and grip strength was not sufficient at the time of 3 months after surgery. In conclusion, patients submitted to pancreatic cancer surgery observed after 3 months of recovery had no improvements in physical function or QOL. It was observed that that the physical function conditions contributed negatively to QOL

## Figures and Tables

**Table 1:** Characteristics of the study subjects.

Parameters	
Sex (male/female) ^a^	8/12
Age (y) ^b^	69.8 ± 7.4
Height (cm) ^b^	156.8 ± 9.0
Comorbidities ^a^	
Bronchial asthma	1
Interstitial pneumonia	1
Diabetes mellitus	3
Hypertension	1
Knee Osteoarthritis	1

^a^: number, ^b^: Mean ± standard deviation.

**Table 2:** Factors predicting of QOL at three months after surgery.

Item	Included Variable ^†^	B	Standard. Error	β	t	**R**	**Adjusted** R^2^	*p* Value
GHS	Grip strength three months after surgery	1.834	0.579	0.598	3.166	0.598	0.322	*p* < 0.05

^†^ Variables were selected by backward stepwise multiple regression models; GHS, Global health status; B, unstandardized Coefficient; β, standardized coefficient.

## Data Availability

The data presented in this study are available on request from the corresponding author. The data are not publicly available due to Participant’s personal information.
